# 基于混合模式的高效液相色谱固定相研究进展

**DOI:** 10.3724/SP.J.1123.2025.06016

**Published:** 2026-06-08

**Authors:** Shijie LI, Zhen LI, Zixia TANG, Mei LYU, Litao WANG

**Affiliations:** 1.滨州医学院，山东 烟台 264003; 1. Binzhou Medical University，Yantai 264003，China; 2.济宁医学院，山东 济宁 272000; 2. Jining Medical University，Jining 272000，China

**Keywords:** 高效液相色谱, 混合模式, 固定相, 新型材料, 综述, high performance liquid chromatography （HPLC）, mixed-mode, stationary phase, new materials, review

## Abstract

高效液相色谱（HPLC）是现代分析化学中一种重要的分离分析技术，广泛应用于生物化学、医药和食品等领域。然而，随着样品复杂性的增加，传统单模式色谱难以实现混合物的高效分离。为此，混合模式色谱（MMC）通过整合反相液相色谱（RPLC）、亲水作用色谱（HILIC）及离子交换色谱（IEC）等多种分离机制，结合功能化固定相，实现了对复杂样品的高效分离。本文简要描述了4种混合模式的分离机理，详细总结了固定相化学反应类型、填料结构以及新型固定相材料，系统综述了2020-2024年基于RPLC/IEC、RPLC/HILIC、HILIC/IEC及RPLC/HILIC/IEC 4种混合模式的高效液相色谱固定相的应用，并分析了不同模式在复杂样品分离中的优势与局限性。最后，本文展望了混合模式固定相在高效液相色谱应用中所面临的挑战及未来的发展趋势。

## Abstract (en)

High performance liquid chromatography （HPLC） remains a core separation and analytical technique in modern analytical chemistry. Boasting prominent advantages including high quantitative accuracy， rapid analysis speed， strong selectivity， and high sensitivity， it has deeply permeated key fields such as biochemistry， pharmaceutical R&D， food testing， environmental monitoring， and materials science， delivering crucial support for the accurate qualitative and quantitative analysis of target components in complex systems. However， with the increasing complexity of analytical samples in scientific research and industrial production， marked by enhanced matrix interference， an expanded polarity range of target compounds， and widespread coexistence of multiple components， the limitations of traditional single-mode chromatography （e.g.， reversed-phase liquid chromatography （RPLC）， hydrophilic interaction chromatography （HILIC）， ion-exchange chromatography （IEC）） have become increasingly pronounced. Specifically， RPLC exhibits insufficient retention and separation capabilities for highly polar compounds， HILIC struggles to handle hydrophobic substances effectively， and IEC is only applicable to the separation of ionic components. None of these single-mode techniques can meet the demand for efficient and comprehensive separation of complex samples. To address this technical bottleneck， mixed-mode chromatography （MMC） has emerged as a viable solution. Its core innovation lies in integrating two or more separation mechanisms into a single chromatographic column. Through the synergistic effects of functionalized stationary phases， MMC enables efficient separation and accurate analysis of complex systems， thereby significantly expanding the application scope of HPLC. This paper briefly elaborates on the separation mechanisms of four mainstream mixed modes， namely RPLC/IEC， RPLC/HILIC， HILIC/IEC， and RPLC/HILIC/IEC. It also summarizes in detail the key chemical reaction types for stationary phase preparation （e.g.， click chemistry reactions and free radical polymerization reactions）， typical packing structures， as well as the characteristics and preparation strategies of novel functional materials such as porous organic cages （POCs）， metal-organic frameworks （MOFs）， covalent organic frameworks （COFs）， carbon quantum dots （CQDs）， microporous organic networks （MONs）， and ionic liquids （ILs）. Based on the latest research findings from 2020 to 2024， this paper systematically reviews the application cases of the aforementioned four mixed-mode stationary phases in practical scenarios such as traditional Chinese medicine component analysis， environmental pollutant detection， food quality control， and pharmaceutical research and development. It also conducts an in-depth analysis of the technical advantages of these four mixed modes， as well as the limitations of some stationary phases， including insufficient stability under extreme pH conditions， complex preparation processes， and high costs for large-scale production. Finally， this paper outlines the core challenges currently confronting mixed-mode stationary phases， including cumbersome synthesis steps， easy degradation and inactivation of functional groups， and difficulties in mobile phase optimization. It further points out that future development trends should focus on simplifying preparation processes， developing environmentally friendly and smart responsive materials， and enhancing the feasibility of large-scale production. The aim is to provide theoretical reference and technical support for the design and development of novel high-efficiency stationary phases， and facilitate greater breakthroughs in mixed-mode chromatography technology in the field of complex sample separation.

高效液相色谱（HPLC）作为现代分析化学中不可或缺的关键技术^［1］^，凭借其高定量精度、分析速度快、高选择性以及高灵敏度等优势，已广泛应用于化学、医药和食品等领域^［2-4］^。传统的单模式液相色谱，如亲水作用色谱（HILIC）、反相液相色谱（RPLC）和离子交换色谱（IEC）用于各种样品分离分析^［5］^。然而，随着实验样品复杂程度的增加，传统单模式液相色谱很难实现混合物高效分离^［6］^。例如，RPLC特别适合于分离非极性和极性较弱的化合物，但对于极性较高的化合物，其分离和检测能力有限^［7］^；HILIC在分离极性和亲水性化合物方面表现出色，但在处理疏水性化合物时效果不佳^［8］^；IEC则主要用于不同基质样品中离子物质的分离分析^［9］^。为了突破传统单模式液相色谱的局限，研究者们积极探索创新，开发出融合多种分离机制的混合模式色谱（MMC）。这种技术指在一根色谱柱中整合两种或以上分离机制，通过协同作用提升复杂样品分离效能^［10］^，从而显著提升了对复杂样品的选择性，拓展了HPLC在复杂样品分析中的应用范围^［11］^。

固定相作为MMC的核心组成部分，其性能直接决定分离效率的高低。因此，开发兼具多种作用机制的新型固定相，已成为该领域的研究热点与核心方向。传统的固定相填充材料主要由C18、C8、氰基和二醇等基团键合在硅胶（SiO_2_）表面制得，在分析目标较为简单时表现出优异的分离性能。但随着分析样品逐渐复杂，这些填料出现酸碱不稳定和使用寿命短等问题。例如，SiO_2_填料在pH大于8时表面的硅羟基使碱性化合物峰形拖尾，影响分离效果；pH小于2时，键合相容易水解断裂，限制其在极端条件下的应用^［12］^。随着MMC技术的发展，新型固定相材料如多孔有机分子笼（POCs）^［13］^、金属有机框架（MOFs）^［14］^、共价有机框架（COFs）^［15］^、碳量子点（CQDs）^［16］^、微孔有机网络（MON）^［17］^和离子液体（ILs）^［18］^等被设计和开发，显著拓展了MMC的应用范围。以SiO_2_为载体材料，与其他功能材料或基团相结合，制备具有特定分离特性的MMC固定相，从而实现对复杂样品的高效分离。这种固定相通过巧妙的材料组合和结构设计表现出多种作用机制，如亲水作用、疏水作用、氢键作用、偶极-偶极相互作用和*π-π*作用等。这些多重作用机制的协同效应极大提升了MMC分离的选择性、效率和稳定性。

MMC根据溶质与固定相的作用力不同，可分为RPLC/IEC、RPLC/HILIC、HILIC/IEC和RPLC/HILIC/IEC等4种模式^［19］^。目前已有综述系统梳理了2016-2020年间混合模式固定相的研究进展^［5］^，鉴于混合模式色谱固定相在近几年呈现快速发展态势，新型材料与制备技术不断涌现，因此本文聚焦2020-2024年期间发表的研究进展。根据上述4种混合模式，综述了近5年来以SiO_2_为载体的固定相在4种混合模式中的应用，并展望了其在HPLC应用领域的未来发展趋势与挑战，以期为新型固定相的设计和开发提供新的思路。

## 1 固定相的设计与制备

### 1.1 固定相化学反应类型

在HPLC中，固定相表面基团决定其适用的混合模式类型，而通过不同化学反应类型制备的固定相直接影响其结构与性能，进而影响其适用的混合模式。常见的化学反应有点击化学反应（巯基-烯点击化学）和自由基聚合反应等。

#### 1.1.1 点击化学反应

点击化学反应凭借其高效性和选择性，成为构建多功能固定相的常用手段。其优势在于能够精准引入功能基团，灵活调控固定相的多模式保留特性，进而增强固定相的稳定性和重现性。例如，Zhang等^［20］^通过点击化学反应，将长链离子液体衍生的CQDs键合至巯基改性的SiO_2_表面，合成了固定相SiO_2_-MPS-CQDs。该材料结合了CQDs的亲水作用、咪唑阳离子基团离子交换作用和长碳链的疏水作用，在RPLC/HILIC/IEC模式下，能够高效分离多种化合物，包括烷基苯、多环芳烃（PAHs）、核苷碱基、磺胺类药物和阴离子化合物等。经过长时间使用和不同pH流动相条件下，分离性能几乎不变，表明其具有良好的重复性和稳定性。此外，在实际应用中成功分离出苹果中的碱基核苷，证明了其在实际样品分析中的应用潜力。Luo等^［21］^通过自由基介导的巯基-烯点击化学合成了Sil-AVI-ST固定相。该材料结合了亲水和疏水等多种作用协同效应，实现了亲水性和疏水性物质的高效分离。与传统的RPLC色谱柱相比，该固定相能够在富水条件下对疏水性分析物进行检测，克服了传统RPLC柱的局限性。

#### 1.1.2 自由基聚合反应

自由基聚合反应是接枝高分子链的理想选择，可为固定相引入多样化的相互作用位点。其核心优势在于能够设计共聚物结构，借助单体比例的调整，精准调控固定相的亲水与疏水特性，从而灵活应对复杂样品的分离挑战。例如，Wang等^［22］^通过原子转移自由基聚合技术，将含有苯环和氰基的咪唑盐单体接枝到SiO_2_表面制备Silica-PILs固定相。利用咪唑阳离子上的苯环和氰基提供*π-π*、疏水和离子交换作用，成功分离了5种非甾体抗炎药（NSAIDs）。并在pH 3.0~9.0的流动相中经过300多次分离后，保留时间和分离效率保持稳定，表现出良好的稳定性和重复性。Wang等^［23］^基于*S*-（4-乙烯基苄基）半胱氨酸（SVC），通过聚合反应与SiO_2_键合，得到固定相Sil-SVC。将疏水性单体*N*-（4-苯基丁烷-2-基）丙烯酰胺（NPA）引入Sil-SVC中制备了另一种固定相Sil-SVC-NPA。结果表明，两种固定相均展现出混合模式色谱的特性，能够通过多种相互作用实现对疏水性化合物、亲水性核苷和碱基以及无机阴离子的高效分离，柱效高达60 000 plates/m。SVC是两性离子基团，可以提供离子交换、氢键和亲水作用。通过引入疏水单体NPA，可以调节固定相的疏水性能，增强其在RPLC模式下的分离能力，从而优化分析物的分离效果，为混合模式色谱提供了新策略。Wang等^［24］^通过自由基聚合反应一锅法制备了聚（苯乙烯-丙烯酸）共聚物改性的硅基固定相SiO_2_@P（St-AA）（图1）。共聚物中苯环结构提供了疏水和*π-π*作用，烷基苯和多环芳烃在固定相上的保留随着流动相中甲醇含量的增加而增强；而羧酸基团则导致极性分析物的保留随乙腈（ACN）含量的增加而减弱。与C18和Amide固定相相比，SiO_2_@P（St-AA）在RPLC和HILIC模式下均表现出良好的分离性能。通过调节流动相的pH值，可以显著影响固定相对有机碱和有机酸的阳离子交换能力，从而优化色谱分离效果，为新型聚合物改性硅基固定相的发展提供了一种新思路。

**图1:**
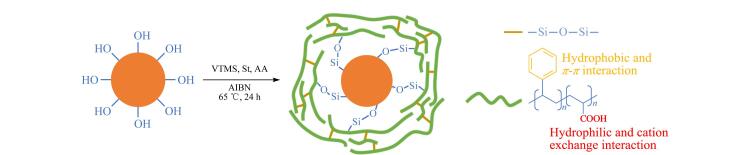
一锅法制备SiO_2_@P（St-AA）^［24］^

### 1.2 固定相填料结构类型

填料的结构类型对色谱性能有重要影响，目前较为常见的填料结构类型为核-壳型。核-壳型填料具有柱压低、负载量大、分离速度快、分离效率高等优点，在HPLC快速分离分析方面具有显著的优势。Shen等^［25］^通过物理涂覆，将聚乙撑亚胺（PEI）和醇溶蛋白（Zein）共同修饰在SiO_2_表面，制成了核壳型固定相PEI/Zein@SiO_2_。Zein是含疏水和含硫氨基酸的生物蛋白，具成膜性和疏水性；PEI是热稳定聚合物，含高密度可修饰端基，能提供亲水相互作用。两者结合在HILIC和RPLC模式下均展现出良好的分离性能和选择性，该柱在酸性条件下具有良好的稳定性，但在碱性条件下稳定性较差。Chen等^［26］^以SiO_2_为核心，2，4，6-三甲酰基间苯三酚（Tp）和二羟基苯胺（BD-（OH）_2_）为壳，制备了SiO_2_@TpBD-（OH）_2_。在制备过程中，先用3-氨基丙基三乙氧基硅烷（APTES）处理SiO_2_，再在其表面生长共价有机聚合物（COPs）壳层，最后通过共聚反应形成核壳结构。通过控制Tp和BD-（OH）_2_的用量，调节COPs壳层厚度，进而改变固定相的亲水性和疏水性。COPs壳层中丰富的羟基、羰基、亚胺、环己基和苯环等基团提供了多种相互作用位点，能够有效分离苯同系物、PAHs、核苷碱基及酸性有机化合物，展现出广泛的分离范围。

### 1.3 新型固定相材料

不同混合模式取决于固定相材料的表面性质，根据固定相材料表面基团与分析物作用力的不同，实现不同物质的分离与保留。目前新型固定相材料有如下几种。

#### 1.3.1 多孔有机分子笼

POCs是一类具有高度有序孔隙结构的有机材料，其孔径和孔道可调节，能够高效吸附和分离小分子及离子。结构中的苯环、环己烷、羟基和伯胺基团赋予其疏水性、亲水性、氢键和*π-π*相互作用，在RPLC和HILIC模式下展现出良好的分离效果。通过引入不同基团，可进一步增强在不同混合模式色谱下对各种化合物的分离能力。本课题组^［27］^基于RCC3分子笼（图2a）将C10通过共价键修饰固定在SiO_2_表面，制备了固定相RCC3-C10@silica（图2b）。RCC3和C10基团与分析物发生多种相互作用，实现非极性至极性化合物的广泛分离，并在纯水体系中有效分离核苷化合物，符合绿色化学原则。此外，RCC3分子笼的手性微环境使其在手性分离中表现出色。2024年本课题组^［28］^通过在RCC3-SiO_2_表面键合环氧丙醇，合成了RCC3-GLD@silica固定相（图2b）。环氧丙醇引入的羟基和氨基增强了固定相的极性，使其在RPLC/HILIC模式下展现出优异的分离性能，尤其在分离酸性化合物、异构体和黄酮类药物时表现出高选择性和分离效率，优于C18柱和商业二醇键合硅胶柱。同年，本课题组^［29］^基于RCC3@SiO_2_引入氰基官能团，合成了固定相RCC3-CN@SiO_2_（图2b）。氰基的引入为固定相提供了亲水、氢键等多种相互作用机制，增强了对不同类型化合物的保留和分离能力。该固定相在RPLC和HILIC模式下均表现出良好的分离性能，适用于从弱极性到强极性化合物的广泛分离，并可在纯水系统中高效分离核苷类化合物，符合绿色分析化学原则。

**图2:**
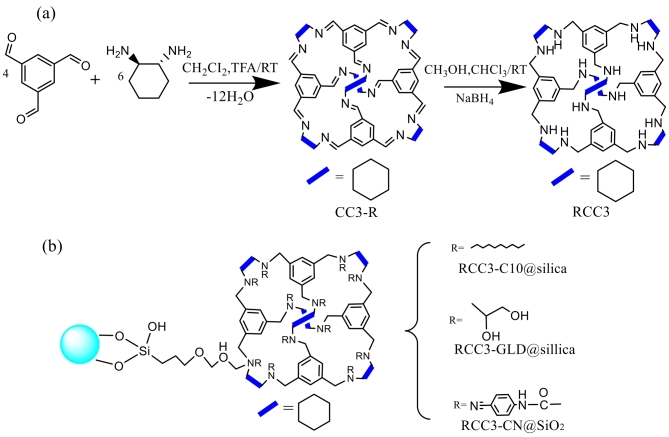
（a） RCC3分子笼制备流程；（b）3种不同分子笼固定相结构示意图^［27］^

#### 1.3.2 金属有机框架

MOFs是由金属离子或簇与有机配体自组装形成的晶态多孔材料，因独特的物理化学特性，在混合模式色谱中具有广泛的应用前景。其可调节的孔隙结构和丰富的功能基团使其能够适应不同的色谱分离模式，并在复杂样品分析中展现出优异的性能。Si等^［30］^基于2D MOFs和ILs，制备了2D Cu-BDC/IL@silica。在HILIC模式下成功分离6种核苷和核碱基，在RPLC模式下分离苯甲酸衍生物、PAHs和烷基苯等疏水性物质。ILs的高亲水性使其在分离亲水性化合物时表现出更高的选择性和分离效率，优于2D Cu-BDC@silica、IL@silica、NH2SPS 100-5和裸SiO_2_柱，为2D MOFs在色谱领域的应用提供了新思路。Si等^［31］^通过在SiO_2_表面修饰L-半胱氨酸（L-Cys）并与缺陷型MOF-919复合，制备了d-MOF-919/cys@silica固定相。在HILIC模式下，该固定相对10种碳水化合物、8种磺胺类药物和8种核苷/碱基混合物展现出卓越的分离效率与选择性；在RPLC模式下，对PAHs和苯甲酰脲（BUs）等疏水性化合物也能高效分离。其色谱重现性和稳定性出色，不同批次间相对标准偏差（RSD）小于1.81%，为MOFs在色谱分离领域的应用提供了新思路和重要参考。Si等^［32］^将聚丙烯酰胺（PAM）与缺陷介孔MOF-818结合，修饰在SiO_2_表面，制备了d-MOF-818/PAM@silica固定相。该固定相在RPLC/HILIC模式下能有效分离多种亲水性和疏水性分析物，如9种核苷和碱基、8种生物碱、6种抗生素和5种烷基苯，且成功应用于金银花活性成分的分离，展现出广泛的应用潜力。其保留时间和柱效的RSD分别小于0.8%和0.9%，表明该固定相在色谱分离中具有较高的可靠性和重复性。

#### 1.3.3 共价有机框架

与POCs、MOFs类似，COFs作为一种有机多孔材料，凭借其独特的结构和孔道表面化学官能团，能够对目标分子进行选择性吸附。它具备多种相互作用力，展现出混合模式色谱特性，可高效分离各类溶质。其结构与性能的可调控性，使其在复杂体系的分离分析中具有广阔的应用前景。Long等^［33］^以2，4，6-三（4-氨基三苯基）-1，3，5-三嗪（TAPT）和TP为原料，通过席夫碱反应键合至SiO_2_表面，合成固定相TAPT-TP-COF@SiO_2_。该固定相利用多种官能团（包括羟基、氨基、醛基、三嗪和芳香环）的协同效应，提供了多种保留机制，实现了对多种极性和非极性化合物的高效分离。在RPLC模式下，能够快速分离直链烷基苯和邻苯二甲酸酯类化合物；在HILIC模式下，该固定相对核苷、磺胺类药物和维生素等极性化合物展现出良好的分离效率。与传统的C18柱相比，TAPT-TP-COF@SiO_2_在分离极性化合物时表现出更优越的性能，为HPLC分离提供了一种新的高性能材料。Wei等^［34］^通过在低共熔溶剂（DES）中进行原位生长反应，将聚丙烯酸（PAA）和COF依次修饰到SiO_2_表面，制备了固定相PAA/COF@SiO_2_。PAA的加入提供了多种相互作用力，如氢键、静电、亲水、疏水和*π-π*相互作用，从而实现了对极性和疏水性化合物的高效分离。该固定相不仅成功分离了多种核苷类化合物，表现出良好的亲水性分离能力，还对烷基苯、PAHs、芳香酮等疏水性化合物实现了基线分离，保留时间与化合物的疏水性和*π-π*共轭系统的大小呈正相关。与COF@SiO_2_、PAA@SiO_2_和SiO_2_柱相比，PAA/COF@SiO_2_柱在分离亲水和疏水化合物时表现出更高的柱效和更好的分离选择性。在实际应用中，该固定相成功检测了环境水样中的烷基酚，表明其在复杂基质中具有良好的抗干扰能力和实际应用潜力。Xia等^［35］^以SiO_2_为核心，通过共价连接Tp和对苯二胺（Pa-1）构建COF-TpPa-1作为外壳，制备了固定相COF-TpPa-1@SiO_2_。与传统的C18柱相比，COF-TpPa-1@SiO_2_柱在分离不同极性分析物时展现出更高的分辨率、稳定性和重复性。并实现了土壤中5种苯脲除草剂残留物的有效检测，显示出在实际样品分析中的潜在应用价值。

#### 1.3.4 碳点/碳量子点

碳点（CDs）材料因其具有较小的粒径、丰富的表面官能团、良好的生物相容性和良好的吸附性能，在色谱分离中展现出广泛的应用潜力。其表面丰富官能团可提供氢键、静电相互作用等多种作用力，使其在混合模式色谱下，能高效分离极性、非极性、亲水性和疏水性等物质，展现出卓越的分离性能。Fu等^［36］^以磷掺杂的碳点（P-DESCDs）为基础，利用三甲氧基硅烷作为桥接剂接枝到SiO_2_表面，制备Sil-P-DESCDs固定相。磷掺杂显著提高了固定相的亲水性和静电相互作用，增强了HILIC模式下对强极性化合物的分离效果。并且该固定相成功应用于中药黄芪丸中毛蕊异黄酮葡萄糖苷的定性和定量分析，检测含量为0.02 mg/mL（图3），验证了其在复杂样品分析中的应用潜力。Chen等^［37］^对CDs作为色谱分离材料的研究进展进行了综述，详细阐述了CDs在多种色谱技术中的应用情况。CDs凭借其独特的物理化学性质，在色谱分离领域展现出显著的性能提升潜力。具体而言，CDs在液相色谱固定相的研究中得到了广泛的应用，并且对色谱分离性能的提升起到了关键作用。这进一步凸显了CDs作为色谱分离材料的巨大潜力。CQDs作为CDs的子集，目前在色谱分析领域也广泛应用。Wu等^［38］^利用CQDs亲水性的特点，引入1，8-二氨基辛烷制备两亲性CQDs，并修饰SiO_2_制备CQDs/SiO_2_固定相。该固定相在不同有机溶剂比例下可实现从RPLC到HILIC的模式转变，对非极性、极性和芳香族化合物具有良好的分离性能。在酸碱条件（pH 3.0和8.0）及不同温度（30、40、50 ℃）下连续运行80 h，色谱性能稳定，RSD小于0.46%，表现出良好的稳定性和重复性。

**图3:**
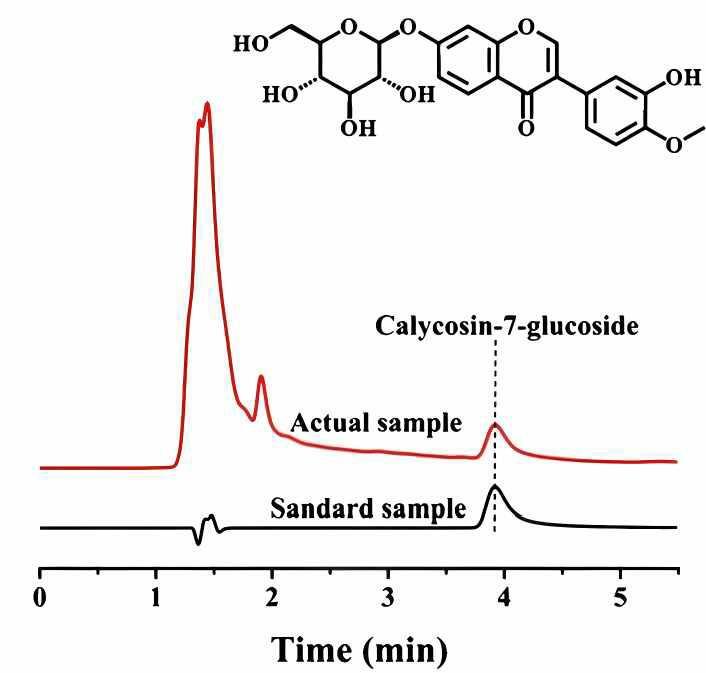
毛蕊异黄酮葡萄糖苷标准品与真实样品分离的色谱图^［36］^

#### 1.3.5 微孔有机网络

MON是一类通过有机合成构建的具有微孔结构的材料，其微孔结构可调节，具有高度的化学稳定性和选择性，适用于分离极性化合物和复杂样品分析。Sun等^［39］^基于MON的巯基-炔点击化学合成了MON-2COOH@SiO_2_，MON中的咪唑结构提供疏水和*π-π*作用，羧基基团的引入显著增强了固定相的亲水性。在分离磺胺类药物、脱氧核苷、生物碱和内分泌干扰化学物质等多种极性和非极性化合物方面表现出良好的分离度，展示了其在混合模式色谱中的潜力。此外，作者还提出了未来的优化方向，并在同年通过引入巯基琥珀酸（MER）制备了固定相MON-2COOH@SiO_2_-MER^［40］^。MER的引入增强了亲水作用，并提供了多个氢键受体位点，改善了HILIC模式下的分离性能。在分离胃丁胺时，该材料的理论塔板数达到27 556 plates/m，高于MON-2COOH@SiO_2_柱（14 536 plates/m），显著提高了柱效。

#### 1.3.6 离子液体

ILs具有独特的理化性质和丰富的阴阳离子种类，其阴阳离子的多样性可提供氢键、静电相互作用、疏水作用等多种作用力，结合其独特的离子液体特性，使其在RPLC、HILIC和IEC模式中展现出混合模式色谱特性，能高效分离极性、非极性、亲水性、疏水性和离子型等各类化合物，使其在复杂体系分离分析中展现出广阔的应用前景。Ji等^［41］^通过分步键合反应制备了一种咪唑基双阳离子离子液体（DILs）和十八烷基共同修饰的固定相（Sil-C18-IL-C4）。Sil-C18-IL-C4固定相在RPLC、HILIC模式下均展现出良好的分离性能。特别是在HILIC模式下，对6种碱基核苷类化合物能够实现完全分离。同时，该固定相还具有良好的分离重复性，保留时间的RSD为0.27%~1.04%，表明其在复杂样品分析中具有良好的应用潜力。Wang等^［42］^和Wu等^［43］^分别通过改变阴离子类型制备了基于SiO_2_聚合咪唑ILs的固定相PIm-Br/coPIm-VBS和Sil-IM-Br/Sil-IM-SAG。在RPLC和HILIC模式下，这些固定相均展现出优于传统固定相的分离性能，主要得益于其增强的离子相互作用、氢键作用以及疏水性。然而，在IEC模式下，由于阴离子的静电排斥作用，coPIm-VBS和Sil-IM-SAG的分离效果均有所降低。总体而言，通过改变阴离子类型制备的固定相在混合模式下表现出良好的分离效果。基于此，Yang等^［44］^基于DILs开发了一种新型的咪唑液体离子固定相Sil-DCH-Im2。双阳离子的引入使得固定相具有*π-π*、氢键、静电和疏水作用，从而在混合模式下展现出良好的分离性能。在实际样品分析中，Sil-DCH-Im2能够高效检测和定量分析腌鱼中的硝酸盐和亚硝酸盐以及酱油中的山梨酸钾和苯甲酸钠，具有高柱效（91 880 plates/m）、良好的重复性（RSD小于4.02%）和高回收率（95.90%~100.19%）。

## 2 混合模式下的应用

### 2.1 反相/离子交换色谱

RPLC/IEC是一种结合了RPLC和IEC两种分离机制的色谱技术。在这种模式下，固定相同时具备疏水作用位点和离子交换位点，能够根据溶质的疏水作用和离子交换作用实现双重保留机制，常用于分离非极性和离子型化合物。其中IEC又分为阳离子交换色谱（利用负电荷基团吸附阳离子）和阴离子交换色谱（利用正电荷基团吸附阴离子），通过调节流动相的pH值和离子强度，可以优化分离效果，从而高效分离复杂样品。近几年报道的RPLC/IEC混合色谱模式固定相总结于表1。

**表1:** RPLC/IEC混合模式固定相的信息及分离物质

Stationary phase	Functional ligand	Hydrophobic interactions	Ion-exchange interactions	Samples	Ref.
Silica-PILs	1-（4-vinylbenzyl） -3-cyanomethyl imidazolium bromide	benzene ring	imidazole cationic substituent	polycyclic aromatic hydrocarbons， alkylbenzene， nonsteroidal anti-inflammatory drugs	［^22^］
C18/TMA	ODS， 3-glycidoxypropyltrimethoxysilane， TMA	ODS	quaternary ammonium group	non-polar substance， small molecule base， aromatic acid	［^45^］
Sil-PBQA	amino anion exchange groups， benzene rings， and quaternary ammonium salts containing benzyl groups	benzene ring	quaternary ammonium group	nonsteroidal anti-inflammatory drugs	［^46^］
Sil-VDI	VDI， thiol functionalized SiO_2_	alkyl	imidazole cationic substituent	hydrophobic alkylbenzene， polycyclic aromatic hydrocarbons and benzene derivatives， inorganic anions	［^47^］
SiO_2_-UI-PMA	UI， PMA	alkyl， PMA	imidazole cationic substituent	EED， sulfonamide drugs， foodborne stimulants， and inorganic anions	［^48^］
Cys-silica-C*n*， *n*=2， 8 and 18	cysteine and vinyl functionalized SiO_2_， bromoethane， 1-bromooctane， and 1-bromooctadecane	alkyl	quaternary ammonium group， cysteine	6 alkaline proteins， 5 acidic proteins	［^49^］
Sil-peptoids	vinyl modified SiO_2_， three small peptides	hydrophobic carbon chain	-COOH， -NH_2_	acidic， polar and non-polar molecules， nonsteroidal anti-inflammatory drugs	［^50^］

PILs： polymeric ionic liquids； PBQA： phenyl-benzyl quaternary ammonium； IEC： ion-exchange chromatography； ODS： octadecylsilyl； TMA： trimethylamine； VDI： 1-vinyl-3-dodecylimidazole bromide； UI： 2-undecylimidazole； PMA： propyl methacrylate； EED： environmental endocrine disruptingchemicals.

季铵基团在色谱固定相中作为阳离子供体，通过静电吸附作用与阴离子相互作用，可用于反相/阴离子混合模式色谱。Liu等^［45］^通过硅烷化反应在SiO_2_表面先键合十八烷基硅烷（ODS），再引入环氧丙基硅烷，经三甲胺（TMA）改性生成季铵基团（-N⁺（CH_3_）_3_），最终制得C18/TMA固定相（图4）。固定相中十八烷基提供疏水作用位点，并且固定相中的季铵基团提供离子交换位点，实现了非极性物质和有机酸的基线分离，并改善了碱性化合物的拖尾现象。在色谱分析中，反相/阴离子混合模式特别适用于分析酸性药物，但总是受到固定相中残留的硅醇基团影响，导致分析物保留减少和结果重复性差。Xu等^［46］^通过SiO_2_表面的巯基硅烷化处理及巯烯点击反应引入含苯环和苄基季铵盐的强阴离子交换基团制备Sil-PBQA。通过苯环的空间位阻效应减少分析物保留的波动，苄基季铵盐提供稳定的离子交换能力（其离子化状态不受流动相pH的影响），并增强对酸性药物的保留因子，从而提高分析结果的稳定性和重复性。该方法成功分离了5种NSAIDs，并且保留因子保持稳定，每对相邻峰的分辨率均高于1.5。

**图4:**
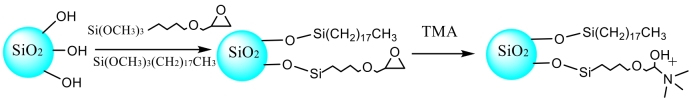
C18/TMA固定相制备流程^［45］^

咪唑基作为RPLC/IEC色谱中的阳离子供体，被广泛地应用于反相/阴离子混合模式色谱固定相中。Li等^［47］^以溴化1-乙烯基-3-十二烷基咪唑（VDI）为配体制备Sil-VDI固定相（图5），通过咪唑阳离子基团和疏水基团的协同作用，实现了对烷基苯、PAHs、苯衍生物、苯胺类物质和无机阴离子的分离分析，且具有良好的分离重现性。此外，固定相在分离肺腺癌细胞膜脂和鸡蛋黄磷脂样品时表现出良好的分离效果，显示出其在磷脂分离分析中的应用潜力。Hu等^［48］^在SiO_2_表面引入2-十一烷基咪唑（UI）和丙基甲基丙烯酸酯（PMA），制备了SiO_2_-UI-PMA固定相。该固定相结合了疏水、离子交换、*π-π*和氢键等多种相互作用，表现出典型的RPLC/IEC混合模式保留行为，成功分离了环境内分泌干扰物（EED）、磺胺类药物、食源性兴奋剂和无机阴离子，相比传统ODS柱展现出更好的分离效果。

**图5:**
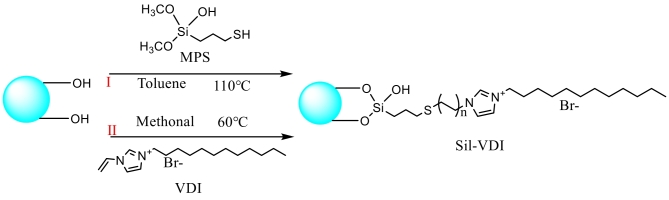
Sil-VDI色谱固定相材料合成示意图^［47］^

在反相/两性离子模式中，通过调控官能团的比例，可以改变阴阳两性离子数量，从而调整固定相与分析物之间作用力的大小。半胱氨酸（Cys）是一种两性离子基团，在不同pH条件下能够与酸性和碱性蛋白质发生不同的相互作用。Wang等^［49］^将Cys和乙烯基官能化的SiO_2_结合，制备了Cys-silica固定相，然后用溴代烷烃（溴乙烷、1-溴辛烷和1-溴十八烷）进一步修饰，得到了功能化固定相（Cys-silica-C*n*， *n*=2、8和18）。这种材料结合了两性离子基团、疏水基团、离子交换基团以及氢键和偶极-偶极相互作用，通过调整流动相pH值和配体的疏水性，实现对复杂样品中酸性和碱性蛋白质的高效分离。Xu等^［50］^通过气相沉积硅烷化方法制备了乙烯基改性的SiO_2_（Sil-Vinyl），然后将3种不同结构小分子拟肽修饰到SiO_2_上，制备了Sil-peptoids固定相。小分子拟肽的加入为固定相引入了氨基和羧基结构，提供了两性离子基团和离子交换作用。该固定相不仅实现了芳烃和抗生素的有效分离，还对极性分子和非极性分子均表现出良好的分离效果，并在不同pH条件下具有良好的选择性和保留能力。

### 2.2 反相/亲水作用色谱

RPLC/HILIC混合模式结合了RPLC的疏水相互作用和HILIC的亲水相互作用。在这种模式下，固定相同时具备疏水和亲水位点，能够根据溶质的疏水性和亲水性差异实现双重保留机制。通过调节流动相的组成和极性，可以优化分离效果，从而高效分离复杂样品中的极性和非极性化合物。近年来，通过引入疏水或亲水基团，RPLC/HILIC混合模式固定相得到了进一步发展（详见表2）。

**表2:** RPLC/HILIC混合模式固定相的信息及分离物质

Stationary phase	Functional ligand	Hydrophobic interactions	Hydrophilic interactions	Samples	Ref.
Sil-AVI-ST	imidazolammonium ionic liquid， stearyl mercaptoacetate， polar ligand	alkyl	imidazole cationic substituent	sulfonamides， vitamins and nucleosides/bases， hydrophobic phthalates， bisphenols， alkylphenols and steroid hormones	［^21^］
PEI/Zein@SiO_2_	PEI， Zein， SiO_2_	Zein	-NH_2_， -COOH	nucleotides， bases， antibiotics， lavonoids	［^25^］
RCC3-C10@silica	RCC3 molecular cage， alkyl functional group	alkyl	-NH_2_， -OH	alkylbenzene， polyphenylene， phenolic， amino， sulfonamide， nucleoside， flavonoid	［^27^］
RCC3-GLD@silica	epoxy propanol， RCC3-R molecular cage	hydrophobic hydrocarbon chain， benzene ring	epoxy propanol group	alkylbenzene， phenols， amines， sulfonamides， nucleosides， amino acids， sugars	［^28^］
RCC3-CN@SiO_2_	RCC3-R molecular cage， -CN	hydrophobic hydrocarbon chain	-CN	alkylbenzene， aromatic， phenolic， amine， sulfonamide， nucleoside， amino acid， carbohydrate	［^29^］
2D Cu-BDC/IL@silica	2D MOFs， ILs	benzene ring	-COOH， ILs	alkaloids， sulfonamides and antibiotics	［^30^］
d-MOF-919/cys@silica	MOF-919， L-Cys	hydrophobic alkyl chain	-OH， -COOH， -SH， -NH_2_	PAHs， BUs， 10 carbohydrates， 8 sulfonamide drugs， 8 nucleoside/base mixtures	［^31^］
d-MOF-818/PAM@silica	MOF-818， PAM	hydrophobic alkyl chain	-OH， -CONH	9 nucleosides and bases， 8 alkaloids， 6 antibiotics， 5 alkylbenzenes	［^32^］
TAPT-TP-COF@SiO_2_	TAPT， TP	hydrophobic alkyl chain	-OH， -NH_2_， -CHO	linear alkylbenzene， phthalates， nucleosides， sulfonamides and vitamins	［^33^］
PAA/COF@SiO_2_	PAA， COFs	hydrophobic alkyl chain	-OH， -NH_2_， -CHO	aromatic ketone， alkylphenol、phthalate esters， PAHs， nucleoside compounds， alkylbenzene compounds	［^34^］
COF-TpPa-1@SiO_2_	Tp， Pa-1， SiO_2_	benzene ring	-OH	non polar alkylbenzene homologues， polycyclic aromatic hydrocarbons， polar amines， phenols	［^35^］
Sil-P-DESCDs	P-DESCDs， （3-aminopropyl）trimethoxysilane	alkyl	-COOH， -OH， -NH_2_	alkylbenzene， polycyclic aromatic hydrocarbons， sulfonamides， aromatic amines， nucleoside bases， and alkaloids	［^36^］
CQDs/SiO_2_	CQDs， 1，8-diaminooctane	alkyl	-COOH， -OH， -NH_2_	14 nucleosides and bases	［^38^］
MON-2COOH@SiO_2_	MON， -COOH， SiO_2_	benzene ring	-COOH	sulfonamide drugs， deoxyribonucleosides， alkaloids and endocrine disrupting chemicals	［^39^］
MON-2COOH@SiO_2_-MER	MON， -COOH， SiO_2_， MER	benzene ring， alkynyl group	-COOH	hydrophobic and hydrophilic probes， sulfonamide drugs， nonsteroidal anti-inflammatory drugs	［^40^］
Sil-C18-IL-C4	C18	C18	imidazole， ILs	nucleoside based compounds， probe molecules	［^41^］
Sil-amide-C11 Sil-C12-amide	amide and hydrocarbon chain groups， 3-aminopropyl	hydrophobic hydrocarbon chain	-CONH	alkylbenzene and polycyclic aromatic hydrocarbons	［^51^］
SiO_2_@COP_Bn-glu_	COP， SiO_2_	benzene ring， alkyl	-COOH	hydrophilic and hydrophobic compounds	［^52^］

HILIC： hydrophilic interaction chromatography； PEI： polyethyleneimine； ILs： ionic liquids； L-Cys： L-cysteine； PAM： polyacrylamide； TATP： 4，4′，4″-（1，3，5-triazine-2，4，6-triyl）trianiline； TP： 2，4，6-triformylphloroglucinol； PAA： polyacrylic acid； Pa-1： *p*-phenylenediamine； P-DESCDs： phosphorus-doped carbon dots； CQDs： carbon quantum dots； MON： microporous organic networks； MOFs： metal-organic frameworks； COFs： covalent organic frameworks； MER： mercaptosuccinic acid； COP： covalent organic polymers； AVI：1-allyl-3-vinyl-imidazolium； ST： stearyl thioglycolate； GLD： glycidyl； PAHs： polycyclic aromatic hydrocarbons； BUs： benzoylureas； Bn-glu： 2，3，4，6-tetra-*O*-benzyl-D-glucopyranose.

Elham等^［51］^在SiO_2_表面引入酰胺和烃链基团，分别制备了Sil-amide-C11和Sil-C12-amide固定相。其中，Sil-amide-C11的酰胺基团嵌入烃链中，表现出较强的非极性保留能力，更适合RPLC模式；而Sil-C12-amide的酰胺基团与烃链分开，具有更强的极性保留能力，更适合HILIC模式。在RPLC模式下，两种固定相可有效分离非极性化合物，但保留能力略弱于C18柱；在HILIC模式下，对极性化合物的保留和分离能力显著，尤其是Sil-C12-amide的自由酰胺基团提供了更强的极性相互作用。通过调整官能团的排列方式，可灵活调节固定相的性能。Li等^［52］^通过四苄基葡萄糖与SiO_2_-苯基发生反应，制备了SiO_2_@COP_Bn-glu_固定相。该固定相因含有苯环和醚键等基团，可与分析物发生*π-π*及电子供体-受体相互作用，显著提升了对各类化合物的选择性。在RPLC模式下，其对芳香化合物和PAHs的分离效果优于C18柱；在HILIC模式下，能有效分离极性化合物（如核苷和核酸碱基），展现出混合模式的保留特性。此外，该固定相还具备良好的重复性、稳定性和立体异构体分离能力，为共价有机聚合物在色谱分离领域的应用开辟了新途径。

### 2.3 亲水/离子交换作用色谱

HILIC/IEC是一种结合了HILIC和IEC两种分离机制的色谱技术。该技术同时提供亲水作用和离子交换作用，常用于分离极性和离子型化合物。近几年报道的HILIC/IEC混合模式固定相总结如下（详见表3）。

**表3:** HILIC/IEC混合模式固定相的信息及分离物质

Stationary phase	Functional ligand	Hydrophobic interactions	Ion-exchange interactions	Samples	Ref.
Sil-PolyCOOH	polyethylene maleic anhydride， SiO_2_-NH_2_	-COOH	carboxyl ionization	nucleosides， nucleic acid bases， carbohydrate compounds， quaternary ammonium salts	［^53^］
（AMPS）*_n_*-ImprSiO_2_	CPTMO， V-Im， AMPS	-CONH， sulfonic acid	imidazole， sulfonic acid	sulfonamide drugs， flavonoids， various components in extracts of traditional chinese medicine ligustrum lucidum	［^54^］
DCC/SiO_2_	DAC， sodium chlorite，SiO_2_-NH_2_	-COOH	-COOH	nucleosides， alkaloids and aniline compounds	［^55^］
C22-A^3^	long chain alkyl， polyamine groups	-NH_2_， -CONH	-NH_2_	nucleosides， nucleotides， aromatic acid compounds， multiple organic and inorganic anions	［^56^］
TPHs-SiO_2_	H3L	-CONH， -NH_2_， -OH	-NH_2_	seven nucleosides and nucleotides， five types of aniline compounds	［^57^］

CPTMO： （3-chloropropyl）trimethoxy silane； V-Im： 1-vinylimidazole； AMPS： 2-acrylamido-2-methyl-1-propanesulfonic acid； DAC： dialdehyde cellulose； H3L： C3-symmetric chiral tricarboxylic acid compound.

Lou等^［53］^通过亲核取代反应将聚乙烯马来酸酐键合到SiO_2_-NH_2_表面成功制备了固定相Sil-PolyCOOH（图6）。该固定相具有亲水、氢键和离子交换多种作用力，实现对核苷、核酸碱基、糖类化合物、百草枯和敌草快季铵盐组分的分离。Ye等^［54］^通过在SiO_2_表面依次接枝氯丙基三甲氧基硅烷（CPTMO）、1-乙烯基咪唑（V-Im）和丙烯酰胺丙磺酸（AMPS）单体，成功制备了多层接枝的（AMPS） *_n_* -ImprSiO_2_固定相。该固定相凭借咪唑、酰胺和磺酸基团，提供了丰富的亲水、离子交换以及*π-π*、氢键等多种作用力，在HILIC/IEC模式下展现出卓越的色谱分离性能。在分离磺胺类药物、黄酮类化合物及中药女贞子提取物中的多种成分时，均实现了良好的基线分离效果，分离选择性显著优于传统C18反相色谱柱。此外，该固定相在不同流动相条件（水含量、盐浓度、pH值和柱温变化）下均表现出稳定的保留行为，充分证明了其在复杂样品分析中的适用性和可靠性。Gao等^［55］^通过将二醛纤维素（DAC）与SiO_2_-NH_2_进行共价偶联，随后利用亚氯酸钠将表面的醛基氧化为羧基，成功制备了DCC/SiO_2_固定相。在HILIC模式下，DCC/SiO_2_通过表面羧基的亲水相互作用，有效分离了核苷、生物碱等强极性化合物，性能与商业GS-120-5-APS柱相当甚至更优。在IEC模式下，DCC/SiO_2_利用表面羧基的阳离子交换作用力，成功分离了多种苯胺类化合物。Zhang等^［56］^通过酰基咪唑介导的酰胺化反应，在SiO_2_表面引入长链烷基和多胺基团，成功合成了C22-A^3^固定相。在HILIC模式下，C22-A^3^凭借其表面多胺基团的亲水相互作用，展现出对核苷、核苷酸和芳香酸等极性化合物的优异分离能力，显著优于传统C18固定相。在IEC模式中，C22-A^3^利用多胺基团与带电分析物的离子交换作用，有效分离了多种有机和无机阴离子。此外，C22-A^3^还表现出对几何异构体、同系物和对映体的显著选择性。Wu等^［57］^通过一锅法合成策略，成功将C3对称的三羧酸手性化合物（H3L）键合到SiO_2_表面，制备了TPHs-SiO_2_固定相。该固定相具有多种作用机制，在HILIC模式下，成功分离了7种核苷和核苷酸，其保留因子（*k*）随流动相中ACN含量增加而显著增大，体现了HILIC模式的典型特征。在IEC模式下，分离了5种苯胺类化合物，其*k*值随流动相pH升高先增后稳，随缓冲盐浓度增加而减小，表明该柱具有典型的离子交换机制。这些结果表明，TPHs-SiO_2_固定相在HILIC和IEC模式下均展现出优异的分离性能，适用于多种极性和离子型化合物的高效分离。

**图6:**
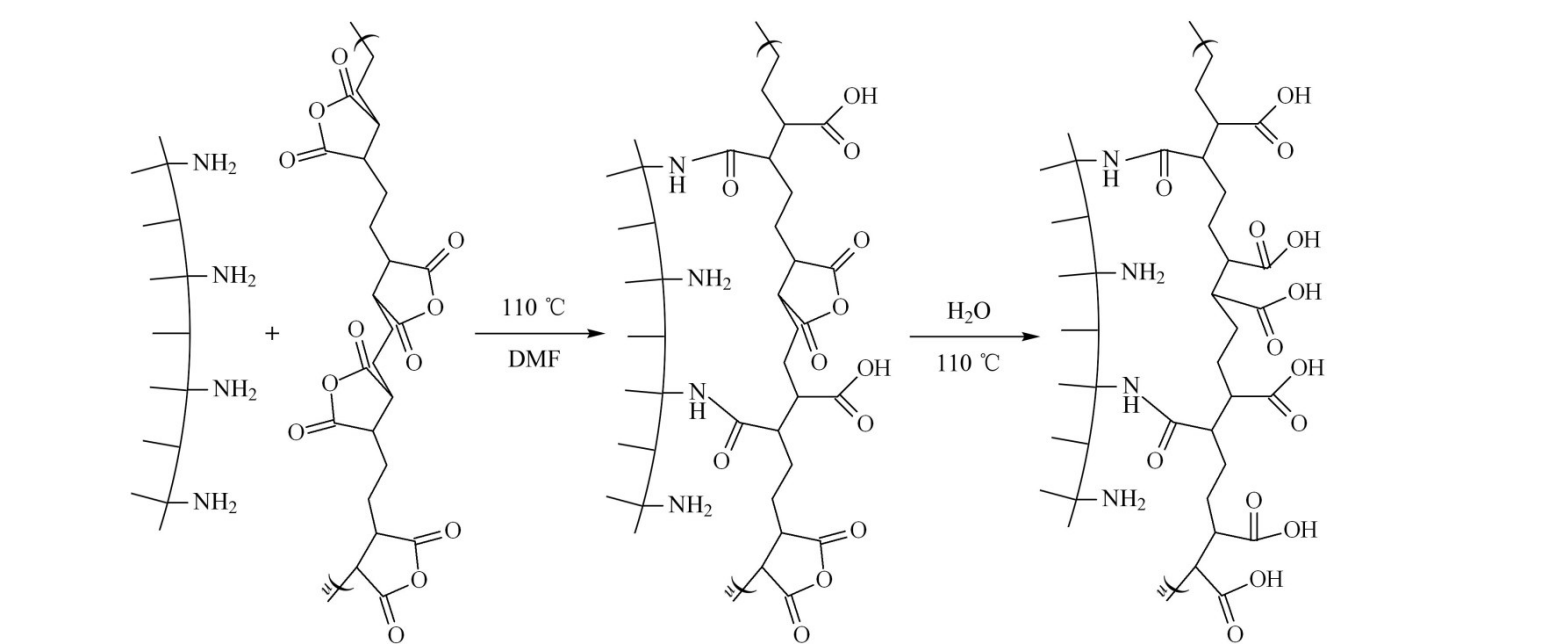
Sil-PolyCOOH固定相合成示意图^［53］^

### 2.4 反相/亲水/离子交换作用色谱

RPLC/HILIC/IEC分离机理通过在固定相表面引入多功能配基，使目标分子与固定相之间同时存在疏水性差异导致的分配作用、极性基团间的氢键或偶极相互作用（亲水作用），以及离子化基团间的静电吸附（离子交换作用）。多种作用力协同下，依据目标分子的疏水性强弱、极性大小及带电状态的不同实现差异化保留与分离。通过精准调控流动相组成（如有机溶剂比例、pH值和离子强度），可实现对宽极性范围及不同离子类型化合物的全面分离，显著提升色谱柱的选择性与适用性。近年来的相关研究进展详见表4。

**表4:** RPLC/HILIC/IEC混合模式固定相信息及分离物质

Stationary phases	Functional ligand	Hydrophobic interactions	Hydrophilic interactions	Ion-exchange interactions	Samples	Ref.
SiO_2_-MPS-CQDs	CQDs， MPS， SiO_2_	alkyl	-OH， -COOH	imidazole cationic substituent	alkylbenzenes， PAHs， nucleoside bases， sulfonamides， anionic compounds	［^20^］
Sil-SVC， Sil-SVC-NPA	SVC， NPA	benzene ring	-COOH， -NH_2_	-NH_2_	hydrophobic analytes， hydrophilic nucleotides， bases and anions	［^23^］
SiO_2_@P（St-AA）	styrene and acrylic acid as polymerization monomers， vinyltrimethoxysilane	benzene ring	-COOH	-COOH	alkylbenzene and polycyclic aromatic hydrocarbons， nucleosides and bases， organic bases and acids	［^24^］
SiO_2_@TpBD-（OH）_2_	COPs， Tp， BD-（OH）_2_	benzene ring	-COOH， -C=N-	imidazole cationic substituent	benzene homologues， polycyclic aromatic hydrocarbons， bases， acidic organic compounds	［^26^］
PIm-Br， coPIm-VBS	SiO_2_ polymerized imidazole ionic liquid	benzene ring， alkyl	imidazole cationic substituent	-COOH， -SO_3_H	nucleosides， inorganic ions and organic ions	［^42^］
Sil-IM-Br， Sil-IM-SAG	IM-Br， IM-SAG， Sil-MPS	benzene ring， alkyl	-OH	imidazole cationic substituent， sulfonate group	organic chemistry， inorganic ions， vitamin	［^43^］
Sil-DCH-Im2	SiO_2_ polymerized imidazole ionic liquid	imidazole ring	-NH_2_， -CONH-	imidazole cationic substituent	hydrophilic substances， hydrophobic substances， acids and inorganic anionic substances	［^44^］
Sil-PEI-APH	PEI， APH	benzene ring	-CONH， -OH	-NHR_2_， -N^+^R_3_	alkylbenzenes， PAHs， nucleosides， flavonoids， aromatic amine	［^58^］
Sil-G1-AS， Sil-G2-AS	BDDE， AS， ethylenediamine	octadecylamine chain	-OH， quaternary ammonium ions	quaternary ammonium ions	PAHs， alkylbenzene， nucleoside， vitamin， phenolic， aromatic amine， flavonoids compounds	［^59^］
DPE-DIL， BND-DIL	DPE， BND	benzene ring	imidazole ring， -CONH， -OH	imidazole cationic substituent	nucleosides and bases， PAHs， food additives	［^60^］

MPS： 3-mercaptopropyltriethoxysilane； SVC： *S*-（4-vinylbenzyl） cysteine； NPA： *N*-（4-phenylbutan-2-yl） acrylamide； BD-（OH）_2_： dihydroxybenzidine； IM-Br： imidazolium bromide； IM-SAG： imidazolium sodium *p*-vinylbenzenesulfonate； Sil-MPS： mercaptopropyl-functionalized silica； APH： *N*-acetyl-L-phenylalanine； BDDE： 1，4-butanediol diglycidyl ether； AS： octadecylamine； DPE： 1，2-diphenylethane-1，2-diamine； BND： *S*-（-）-1，1′-binaphthyl-2，2′-diamine； -N^+^R_3_： quaternary ammonium group.

通过化学键合将带电聚合物链（阳离子和阴离子）接枝到SiO_2_表面，形成具有特定化学性质和分离功能的固定相。Wan等^［58］^将PEI和*N*-乙酰-L-苯丙氨酸（APH）接枝到SiO_2_表面，合成了固定相Sil-PEI-APH。在混合模式下，色谱柱能够有效分离多种不同极性的化合物，包括烷基苯、PAHs、核苷、苯甲酸及其衍生物等。Yang等^［59］^通过在SiO_2_表面依次接枝乙二胺、1，4-丁二醇二缩水甘油醚（BDDE）和十八胺（AS），成功制备了Sil-G1-AS和Sil-G2-AS固定相（后者在前者基础上进一步接枝）。结果显示，Sil-G2-AS固定相具有更强的亲水性，而Sil-G1-AS固定相则表现出更强的疏水性。多次进样测试表明，这两种固定相柱均具有良好的重复性。此外，通过测定盐酸妥洛特罗片剂中的妥洛特罗含量，进一步验证了Sil-G2-AS固定相在实际药物样品分析中的应用价值，表明其可用于药物质量控制和成分分析。Wang等^［60］^以1，2-二苯基乙二胺（DPE）和*S*-（-）-1，1′-联萘-2，2′-二胺（BND）等为原料，通过点击反应引入DILs，并将其键合到SiO_2_表面，制备了DPE-DIL和BND-DIL。前者含有苯环，后者含有联萘基团（提供了更强的*π-π*相互作用和疏水性）。在混合模式下，BND-DIL表现出比DPE-DIL更高的分离性能。

## 3 总结与展望

本文系统综述了2020-2024年基于混合模式的高效液相色谱固定相的研究进展，重点探讨了RPLC/IEC、RPLC/HILIC、HILIC/IEC和RPLC/HILIC/IEC 4种模式的协同分离机制。研究发现，不同模式在复杂样品分析中表现出独特优势与局限性：RPLC/IEC模式适用于分离非极性和离子型化合物（如磺胺类药物与无机阴离子），但对强极性非离子化合物保留不足；RPLC/HILIC模式可覆盖从非极性到强极性化合物（如烷基苯与核苷），但在极端pH条件下固定相稳定性较差；HILIC/IEC模式在分离极性及带电化合物时表现出色，但固定相选择范围有限，且在分离非极性化合物时效果欠佳；RPLC/HILIC/IEC模式兼具3种机制，适用于全极性范围及带电化合物（如黄酮类与有机酸），但其制备工艺复杂，流动相条件优化难度大。在实际应用中，需根据样品特性选择合适的分离模式，生物样品中极性代谢物分析优先采用RPLC/HILIC模式，而环境污染物中多组分共存体系更适合RPLC/HILIC/IEC模式。

通过上述研究表明，混合模式固定相在稳定性、重复性及分离效率方面优于传统固定相，并已在中药成分分析、环境污染物检测和食品检测等实际场景中验证了应用潜力。但目前仍然面临着许多挑战：固定相制备过程中需引入其他功能材料或修饰基团，导致合成步骤复杂、产率低等问题。在实际分离样品时需要在不同流动相条件下切换分离模式，频繁的化学环境改变也会产生固定相中某些功能基团降解或失活等问题。未来的研究方向应着重聚焦于合成工艺的优化。一方面，通过简化合成步骤，提高反应效率，进一步提升目标产物的产率；另一方面，积极探索绿色制备工艺，研发可以降解的材料，减少对环境的影响，降低生产成本。并在此基础上，提升固定相在大规模生产和工业应用中的可行性。此外，还可以结合pH响应材料开发智能响应型固定相。使其能够依据不同流动相条件，自动调整自身表面性质，从而显著提升固定相的重复性与稳定性，为复杂样品的高效分离提供更为可靠的保障。总之，MMC固定相具有很大的发展前景，期待在不久的将来，所面临的挑战都能有所突破和进展，推动固定相技术迈向新的高度，为复杂样品分离提供更加强有力的支持。
